# Potential diagnostic value of N1LR and SNHG1 in acute myocardial infarction

**DOI:** 10.1186/s12920-023-01501-2

**Published:** 2023-04-03

**Authors:** Wei Zhu, Li Luo, Guangning Ye, Jiaman Ou

**Affiliations:** Internal Medicine, Cardiovascular Center, Yangjiang People’s Hospital, Guangdong, China

**Keywords:** Acute myocardial infarction, lncRNA, N1LR, SNHG1

## Abstract

**Introduction:**

Acute myocardial infarction (AMI) is a common cardiovascular disease that can lead to myocardial necrosis and a poor prognosis. Clinical practice requires an accurate and quick diagnosis of AMI due to the inherent limitations of current biomarkers. Therefore, research into novel biomarkers is necessary. We aimed to explore the diagnostic potency of the long non-coding RNA (lncRNA) N1LR and SNHG1 in patients diagnosed with AMI.

**Method:**

We measured lncRNA levels in 148 AMI patients and 50 healthy volunteers with quantitative RT-PCR method. Receiver operating characteristic (ROC) analysis was administered to detect the diagnostic power of selected lncRNAs. Correlation analysis was performed to explore the relationship between N1LR as well as SNHG1 and the conventional myocardial biomarkers (LDH, CK, CKMB and cTnI).

**Results:**

ROC analysis reveals the possibility of N1LR and SNHG1 as biomarkers in AMI diagnosis (AUC of N1LR: 0.873; AUC of SNHG1: 0.890). Correlation analysis revealed that N1LR was negatively correlated with the conventional biomarkers and SNHG1 was positively correlated with the conventional biomarkers.

**Conclusion:**

For the first time, we investigated the potential predictive diagnostic value of N1LR and SNHG1 in AMI diagnosis and substantial outcomes were obtained. Also, they may be capable of reflecting the progress of the disease during clinical practice from the correlation analysis.

## Introduction

Acute myocardial infarction (AMI) is one of the most severe consequences of coronary artery disease and ranks at the leading position of factors causing mortality worldwide [1]. With the increasing adoption of evidence-based management and lifestyle adjustment, the occurrence, as well as mortality of AMI, has considerable reduction in the recent years [2]. However, AMI still occupies a substantial proportion of the global disease burden and causes a huge economic issue to both family and society [3]. In this regard, rapidly accurate diagnosis and treatment are essential to AMI suspicious patients.

Non-coding RNAs have emerged as a potential field for disease diagnosis biomarkers or therapeutic targets, which were verified as important role in cell growth, differentiation, immunity and apoptosis [4, 5]. Long-noncoding RNAs (lncRNAs) are a subclass of RNA transcripts larger than 200 nucleotides in length but without protein transcription capacity. Several studies have proved that lncRNAs are involved in the cardiovascular system, such as serving as transcriptional factors in coronary artery disease, promoting cardiomyocyte proliferation, and involvement in the molecular mechanism of myocardial I/R injury [6–8]. Thus, exploring lncRNA in clinical practice may assist in disease diagnosis and finding the potential therapeutic target. N1LR was initially found to be involved in ischemic stroke via p53 inhibition [9] and upregulation of N1LR was proved to be protective after I/R induced injury via repressing TGF-β1 pathway to inhibit H_2_O_2_-induced apoptosis, inflammatory response and LDH release in cardiomyocyte [10]. SNHG1, another lncRNA, was first identified by its oncogenic promotion in cancer cell proliferation, and an increased level of SNHG1 was associated with decreased survival rate [11]. According to a recent study, it is involved in the phosphatase and tensin homolog (PTEN)/phosphoinositide 3-kinase (PI3K)/protein kinase B (AKT) signaling pathway, and effectively improved function after myocardial infarction [12]. In this regard, we initiated pioneer tests of these lncRNAs for AMI patients, and potentially positive results were found. Consequently, we sought to explore the potential capacity of N1LR and SNHG1 during the clinical practice of AMI, which might aid the diagnosis process of AMI.

In this study, we aimed at examining the potential diagnostic value of N1LR and SNHG1 in AMI diagnosis to provide novel biomarkers that could be used in clinical practice.

## Methods

### Participants

148 consecutive AMI patients presented in Yangjiang People’s Hospital (Guangdong, China) were recruited from January 2021 to July 2022, and a total of 50 volunteers were enrolled as healthy control. The AMI diagnosis is consistent with the 2017 ESC guideline that elevated conventional cardiac biomarkers above the upper limit, and abnormal echocardiogram (ECG) findings [13]. Patients meeting the following criteria were excluded from the study: complicated with other advanced or malignant diseases such as organ failure or cancer. Also, patients who were unwilling to participate in the study were excluded. All enrolled AMI patients were accessed for Thrombolysis in Myocardial Infarction (TIMI) score and Synergy Between Percutaneous Coronary Intervention With Taxus and Cardiac Surgery (SYNTAX) score immediately after diganosis [14, 15]. Healthy volunteers were randomly recruited with the criteria of no history of cardiovascular diseases and other essential organ diseases. After enrollment, blood samples were collected and sent for laboratory tests. For AMI patients, aspirin and clopidogrel were prescribed before a surgery, and, standard percutaneous coronary intervention (PCI) was performed to treat the infarction. After receiving PCI, routine anticoagulant and anti-platelet regimens were prescribed. Informed consent was obtained from all participants involved in this study.

### Blood sample collection

Blood samples were collected in blood collection tubes (BD Vacutainer®, EDTA tubes [367861]) and serum tubes ) when patients were admitted to the hospital. All cardiac biomarkers were measured in the serum level. Lactate dehydrogenase (LDH), creatine kinase (CK), CK-MB and cardiac troponin I (cTnI) were measured by using a Hitachi 7600 automatic biochemical analyzer (Hitachi, Tokyo, Japan) and the corresponding kits (Medicalsystem, Ningbo, China). After being diagnosed with AMI, the remaining blood after blood routine tests were acquired from the EDTA tube without coagulation and hemolysis. The collected peripheral blood samples were centrifuged at 3500 rpm for 10 min and the supernatant was carefully transferred into an RNase-free tube and was eventually frozen at − 80 °C for the following analysis.

### RNA extraction and qRT-PCR

Total RNA was extracted from plasma samples using Plasma/Serum RNA Purification Maxi Kit (Norgen, Product #56200) as described by the manufacturer. iScript® cDNA Synthesis Kit (Bio-Rad) was adopted to perform reverse-transcription of cDNA (component: total 20 μl reaction system containing 200 ng RNA template, 4 μl 5 × iScript Reaction mix, 1 μl iScript Reverse Transcriptase, and Nuclease-free water; reaction protocol: 5 min at 25 °C, 30 min at 42 °C, 5 min at 85 °C, and then hold at 4 °C). RNase-Free DNase I Kit (Norgen, Product #25710) was adopted to on-column DNA removal process to avoid genomic DNA contamination as described by the manufacturer. One Step TB Green® PrimeScript™ RT-PCR Kit II (Perfect Real Time) (cat. no. RR086A; TaKaRa) was administered in qRT-PCR procedure with specifically designed primers for lncRNA N1LR and SNHG1. When performing qRT-PCR of N1LR and SNHG1. GAPDH was treated as the internal control. Specific primers used in this study are as follows: N1LR (forward 5′-TGTGTCAGATGGAACCCTGC-3′ and reverse 5′-AGCACTGTGTGGGTTGAACA-3′), SNHG1 (forward 5′-GCCCACAAGAGCTTACTGGT-3′ and reverse 5′-CACAGCAAACCCTCAACTGC-3′), GAPDH (forward 5′-TGCACCACCAACTGCTTAGC-3′, reverse 5′-GGCAT GGACTGTGGTCATGAG-3′). The relative expression level of detected lncRNA was measured following 2-ΔΔcq methods, with the calibrator of health control plasma. Each sample was tested in triplicate and the mean was calculated for the following analysis.

### Statistical analysis

Values were presented with mean ± SD or number (percentage), and all analyses involved in this study were performed with SPSS (Version 27.0) and R (Version 4.2.1). The following packages were adopted in R software when performing the analyses: *pROC, ggplot2*. For data visualization, R and GraphPad Prism (Version 8.4.2) were adopted. Independent t-test and chi-square test were used to compare the baseline characteristics of the two groups. The receiver operating characteristic (ROC) curve was used to evaluate the sensitivity and specificity of the selected lncRNAs in AMI diagnosis. The area under the ROC curve (AUC) was calculated to evaluate the predictive power of the two selected lncRNAs. Pearson’s test was used to test the correlation among the two selected lncRNAs’ expression levels, the myocardial enzymes (LDH, CK, CK-MB and cTnI) and TIMI score as well as the SYNTAX score. A *P* value less than 0.05 was considered to be statistically significant.

## Results

### Baseline characteristics

A total of 148 AMI patients and 50 healthy volunteers were enrolled in this study. The average age was 68.25 ± 3.29 and 68.60 ± 3.75 for AMI and control group, respectively, and no significance was observed between the two group. Females consisted of 48.6% among AMI group and 54.0% in control group, with no significant difference. The body mass index (BMI) in AMI group was significantly higher than that in control group (27.49 ± 3.37 vs. 21.26 ± 1.57, *P* < 0.001) and, for the risk factors associated with cardiovascular diseases, the percentage of hypertension (63.5% vs. 28.0%) and diabetes mellitus (59.5% vs. 28.0%) were higher in AMI group. For the myocardial enzymes, LDH, CK, CK-MB and cTnI in AMI group were significantly higher than control group. Detailed information about the baseline characteristics was shown in Table 1.

**Table 1:** Baseline Characteristics of AMI patient group and control group

	Control group (n = 50)	AMI group (n = 148)	*P* value
Age (years)	68.60 ± 3.75	68.25 ± 3.29	0.531
Female, n	27 (54.0%)	72 (48.6%)	0.869
BMI (kg/m^2^)	21.26 ± 1.57	27.49 ± 3.37	< 0.001*
Hypertension (n)	14 (28.0%)	94 (63.5%)	< 0.001*
Diabetes Mellitus (n)	14 (28.0%)	88 (59.5%)	< 0.001*
LDH (U/L)	116.50 ± 74.76	257.62 ± 68.50	< 0.001*
CK (U/L)	153.84 ± 55.38	208.86 ± 53.75	< 0.001*
CKMB (ng/ml)	2.55 ± 1.59	5.27 ± 1.43	< 0.001*
cTnI (μg/ml)	0.01 ± 0.01	0.41 ± 0.32	< 0.001*
TIMI score			
Low (0–2)	N/A	13 (8.8%)	
Intermediate (3–4)	N/A	107 (72.3%)	
High (≥ 5)	N/A	28 (18.9%)	
SYNTAX score	N/A	30.45 ± 5.72	

Values are presented in mean ± standard deviation (sd) or n (%)

*BMI* Body Mass Index, *LDH* lactate dehydrogenase, *CK* creatine kinase, *CK-MB* creatine kinase-MB, *cTnI* cardiac troponin I, *TIMI* thrombolysis in myocardial infarction, *SYNTAX* Synergy Between Percutaneous Coronary Intervention With Taxus and Cardiac Surgery

*Indicates significant difference

### The predictive power of N1LR

**Fig. 1:**
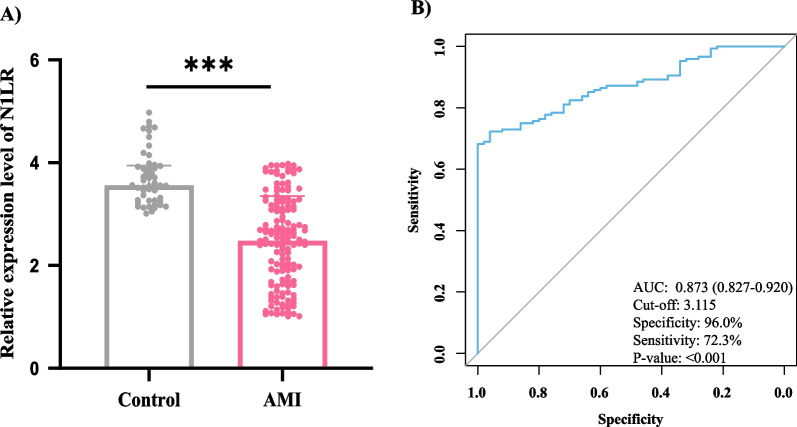
Expression level and ROC analysis of N1LR. **A** Relative expression levels of N1LR were tested in AMI patients and healthy control, and a significant difference was obtained (*P* < 0.05); **B** ROC analysis of N1LR in AMI diagnosis, with the AUC of 0.873 and a cut-off value of 3.115

All enrolled participants’ blood samples were collected to test the N1LR expression level, and the difference was tested utilizing means of Mann Whitney test. Compared with control group, the expression level of N1LR in AMI group was significantly lower (Fig. 1A, *P* < 0.001), which was consistent with the previous animal model [10]. To evaluate the predictive power of N1LR in AMI diagnosis, an ROC curve was drawn. As shown in Fig. 1B, the AUC of N1LR in AMI diagnosis was 0.873 (95% CI 0.827–0.920), with a cut-off of the relative expression level of 3.115, specificity of 96.0%, and sensitivity of 72.3%.

### The predictive power of SNHG1

**Fig. 2:**
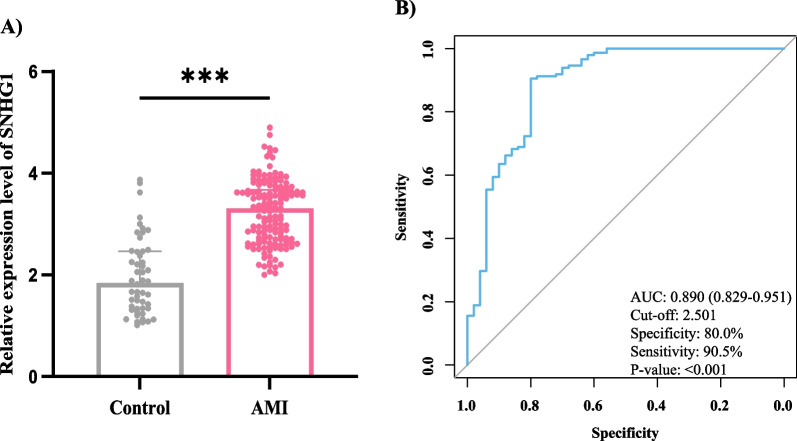
Expression level and ROC analysis of SNHG1. **A** Relative expression levels of SNHG1 were tested in AMI patients and healthy control, and a significant difference was obtained (*P* < 0.05); **B** ROC analysis of SNHG1 in AMI diagnosis, with the AUC of 0.890 and a cut-off value of 2.501

All enrolled participants’ blood samples went through the same process as the abovementioned. Compared with control group, the relative expression level of SNHG1 was significantly higher in AMI group (Fig. 2A, *P* < 0.001), which was consistent with a previous animal model [12]. ROC curve was adopted to evaluate the predictive power of SNHG1 in AMI diagnosis. AUC of SNHG1 was 0.890 (95% CI 0.829–0.951), and the cut-off value of relative expression level was 2.501, with the specificity of 80.0% and sensitivity of 90.5%. Detailed information was shown in Fig. 2B.

### The predictive power of combining N1LR and SNHG1

**Fig. 3:**
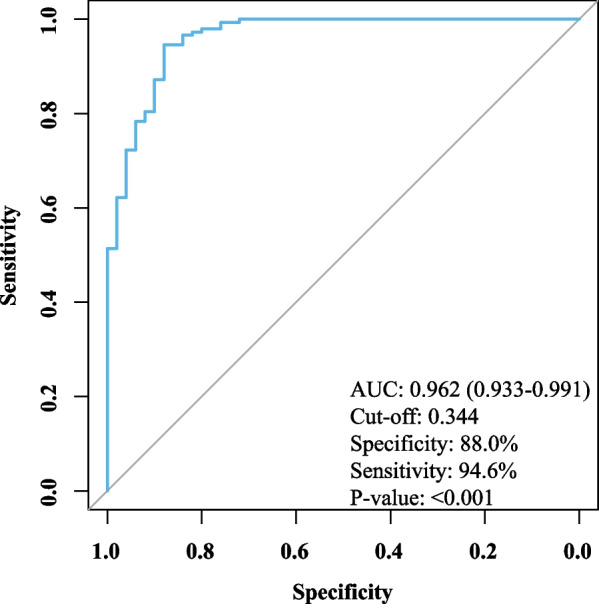
ROC analysis of the two lncRNA. The predictive power of the combination of N1LR and SNHG1 was tested, with an AUC of 0.962

To evaluate whether the predictive power would increase as they were tested alone, we combined the two lncRNAs and performed the ROC analysis. As shown in Fig. 3, the AUC (0.962, 95% CI 0.933–0.991) was higher than either N1LR (0.873, 95% CI 0.827–0.920) or SNHG1 (0.890, 95% CI 0.829–0.951), with the specificity of 88.0% and 94.6%, indicating these two lncRNAs could be potential biomarkers to aid AMI diagnosis.

### Correlation of the selected lncRNAs with myocardial enzymes

**Fig. 4:**
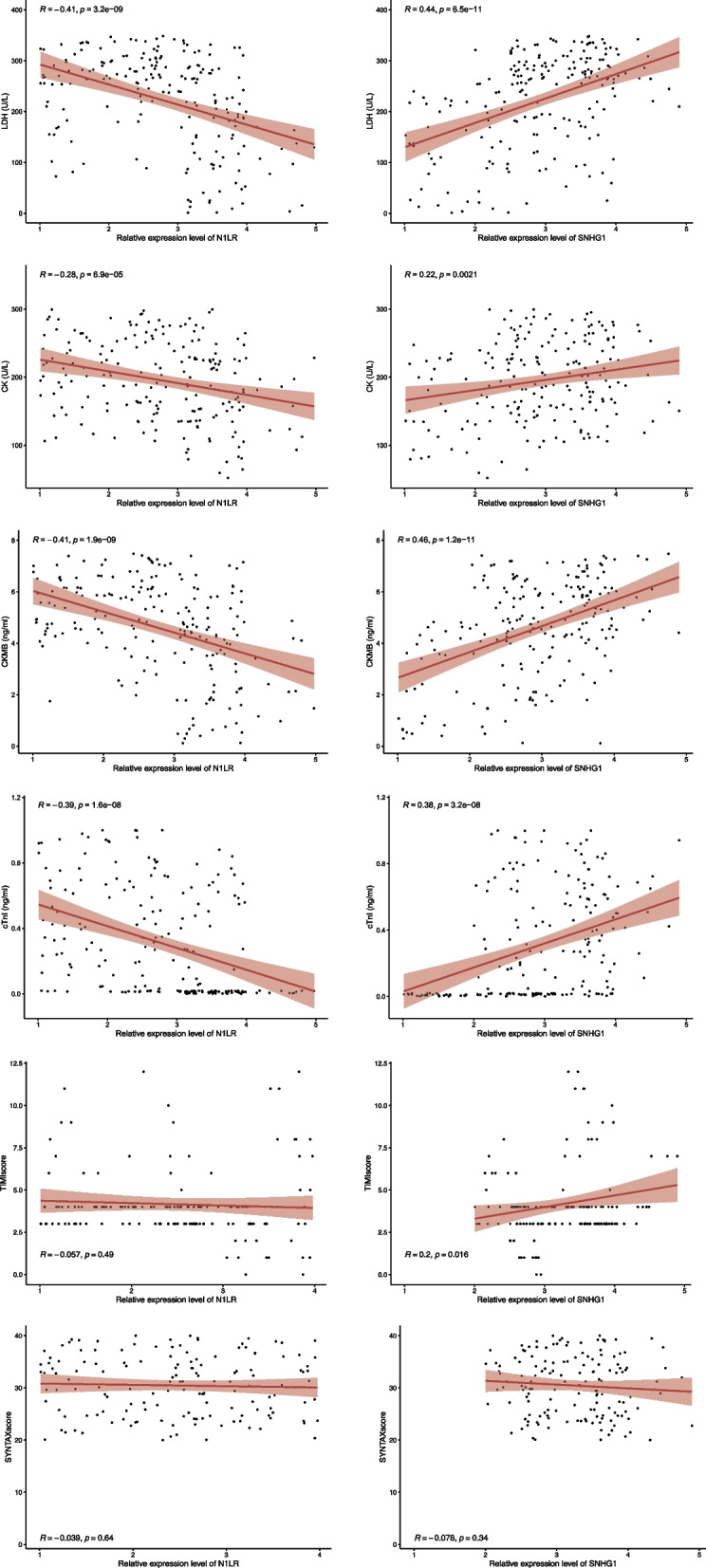
Correlation analysis of the lncRNAs and conventional myocardial biomarkers. The expression level of N1LR was negatively correlated with the conventional biomarkers while SNHG1 was positively correlated with the conventional biomarkers. For the risk score, no correlation between N1LR and TIMI score as well as SYNTAX score. SNHG1 was positively correlated with the TIMI score while no correlation was found with the SYNTAX score

Correlation analysis was performed to investigate the association between the selected lncRNAs with the myocardial biomarkers. For N1LR, it was found to be negatively correlated to the LDH (coefficient = − 0.41), CK (coefficient = − 0.28), CK-MB (coefficient = − 0.41) and cTnI (coefficient = − 0.39), while no significant correlation to TIMI score and SYNTAX score. The correlation is seen to occur when the relative expression level is under four of N1LR. For SNHG1, it was found to be positively correlated to the LDH (coefficient = 0.44), CK (coefficient = 0.22), CK-MB (coefficient = 0.46), cTnI (coefficient = 0.38) and TIMI score (coefficient = 0.20), respectively, while no significant correlation to SYNTAX score. The correlation is seen to occur when the relative expression level of SNHG1 ranges from two to four. The detailed information was shown in Fig. 4.

## Discussion

Rupture of the coronary atherosclerotic plaque resulting in thrombosis is the major reason causing AMI. With continuous increase of infarction size, the coronary artery may be occluded and the blood flow will be disturbed, resulting in systemic circulation disturbance, progressive left ventricular dilation and deterioration of heart function [16]. Increasing studies have proven the potential diagnostic value of lncRNAs in AMI diagnosis [17, 18], and reported that lncRNAs played a significant role in the occurrence of myocardial infarction [19]. To the best of our knowledge, a single biomarker used in a disease’s diagnosis may be inaccurate due to its inherent limitation, such as the multi-pathways involved, yet the combination of several biomarkers may enhance the accuracy of the disease’s diagnosis [20, 21]. Thus, combined with the conventional biomarkers used widely in clinical practice, it may enhance the accuracy and efficacy of AMI diagnosis. In this study, we investigated the potential predictive power of N1LR and SNHG1 in AMI diagnosis and acceptable AUC was obtained via ROC analysis. Besides, we tested the diagnostic value when combining two of them in AMI diagnosis, and a positive outcome was obtained, indicating the possibility of clinical practice administration.

N1LR has been firstly reported as a novel I/R-induced lncRNA and possesses the ability to be neuroprotective in ischemic mice model that overexpression of N1LR could ameliorate the H2O2-induced cell apoptosis, inflammation response, death, and LDH release by prevention of p53 in vitro [8, 10]. Taken together, these results may indicate that N1LR could be a potential factor to protect neurons to ischemic injury. Besides, N1LR was found to be capable of improving cardiac function and alleviating fibrosis as well as inflammation, with the underlying mechanisms of TGF-ß signaling pathway inhibition. For the in-depth mechanism, interestingly, N1LR overlaps the 5ʹ-UTR of protein-coding gene Nck1, and N1LR knockdown leads to increased Nck1 expression [22]. Nck1 has been reported to be involved in cellular remodeling and responding to I/R injury in several ways [23, 24]. However, the current study investigating N1LR is limited, and more studies are needed to explore the molecular mechanism of N1LR.

SNHG1 was found to be involved in cardiac regeneration and repair after myocardial infarction by activating the PTEN/PI3K/AKT pathway and leading to continuous cell cycle re-entry. However, cardiac dysfunction was found under the situation of administrating SNHG1 antagonist [12]. A previous study showed that the loss of PTEN promotes cardiomyocyte proliferation, and SNHG1 can bind to PTEN and enhances this process, which may improve myocardial repair after AMI [25]. In addition, PTEN degradation results in PI3K-AKT pathway activation to promote mammalian cardiomyocyte proliferation and heart regeneration [26]. As a key upstream regulator, SNHG1 modulates AKT phosphorylation and angiogenesis by activating PI3K/AKT pathway.

In previous studies, the relationship between myocardial biomarkers and the progression of AMI has been studied, and it is proposed that the severity of AMI is correlated with the level of these biomarkers [27, 28]. Regarding this, the relationship between the level of the selected lncRNAs and conventional myocardial biomarkers was investigated. As shown in Fig. 4, trends of the LDH, CK, CKMB and cTnI were similar to the expression levels of the lncRNAs. The expression level of N1LR was negatively correlated with the myocardial biomarkers while the expression level of SNHG1 was positively correlated with the myocardial biomarkers. Collectively, the expression level of N1LR and SNHG1 may be capable of reflecting the AMI progression, and it could be verified in future studies.

Several limitations should be addressed in this study. First, expression levels of N1LR and SNHG1 were only tested in AMI patients and healthy volunteers, the situation in patients presented with angina should be verified in future studies. Second, these two lncRNAs may be involved in other pathological processes which may be confounded in complex cases. Third, qRT-PCR is the preferred standard protocol to test the expression of lncRNAs but it is time-consuming and expensive. As a result, it may lead to an adequate economic burden at the beginning, and the popularization of these biomarkers may need a more cost-effective method. Fourth, even with a relatively substantial sample size, predictive accuracy may be improved by a larger sample size. Last, the pathophysiology of these lncRNAs associated with AMI should be explored in future studies.

## Conclusion

In this study, we investigated the potential predictive value of N1LR and SNHG1 in AMI diagnosis for the first time and acceptable outcomes were obtained. In addition, the relationship between the lncRNAs and conventional myocardial biomarkers was tested and similar trends were obtained, indicating the possibility to reflect the progress of the disease. Collectively, the results of this study may aid the rapid as well as accurate AMI diagnosis during routine practice.

## Data Availability

The datasets generated and/or analyzed during the current study are not publicly available due to the privacy of patients, but are available from the corresponding author on reasonable request.
